# Volumetric absorptive microsampling: its use in COVID-19 research and testing

**DOI:** 10.4155/bio-2021-0102

**Published:** 2021-08-31

**Authors:** James Rudge, Stuart Kushon

**Affiliations:** ^1^Neoteryx LLC, 421 Amapola Ave., Torrance, CA 90501, USA

**Keywords:** clinical trials, COVID-19, microsampling, Mitra devices, remote sample collection, SARS-CoV-2, serosurveillance, VAMS technology

## Abstract

COVID-19 led to changes in the way blood samples are collected. As societies were isolated to control viral spread, access to facilities became limited. Remote sample collection with a volumetric microsampling approach, using Mitra^®^ devices based on VAMS^®^ technology, proved to be highly effective. It allowed people to collect high-quality samples at home and post them to a laboratory. This enabled scientists to conduct large serosurveillance studies, with results showing that seroprevalence of COVID-19 was higher than initially expected. Furthermore, remote microsampling studies by several institutions were conducted to measure the relationship between antigen levels and antibody response and duration. VAMS technology was also used in COVID-19 clinical trials. In summary, the independent research reviewed in this paper proved that VAMS is an effective sample collection alternative.

## An unprecedented pandemic

On 11 March 2020, the WHO declared the SARS-CoV-2 outbreak a pandemic, and the world began to change overnight. In the weeks that followed, countries began national lockdowns to halt the spread of the virus which, left unchecked, was threatening to cripple healthcare systems. There was no effective treatment known to the medical community, and no vaccine available. Panic buying ensued and loved ones were isolated from one another as they sheltered at home. The skies became devoid of contrails as airlines were forced to ground most of their fleets and, for many countries, the roads practically emptied as the world watched and waited. Governments began to shore up their resources, stockpiling Personal Protection Equipment and medical equipment. Large, temporary hospitals were opened in venues, such as exhibition centers, to deal with an anticipated tsunami of cases of COVID-19, the infection caused by the SARS-CoV-2 virus.

As COVID-19 cases rose sharply, the need for monitoring the spread of the pandemic became essential. The first tests used to monitor and diagnose active COVID-19 infections were direct antigen tests using polymerase chain reaction (PCR). However, the PCR test using nasopharyngeal swabs had its limitations. While PCR was (and continues to be) useful for diagnosis, this test gives no data on seropositivity. One important reason to measure seropositivity is to ascertain the degree of seroprevalence in a population. Why test for seroprevalence at the beginning of the pandemic? As the crisis continued, it became apparent that not all those who had contracted the illness showed any symptoms and would be less inclined to get a PCR test [1]. Because asymptomatic carriers, or silent spreaders, were not getting tested, this risked under-reporting true historical prevalence rates within a given population and underestimating the proportion of the population that had been exposed to SARS-CoV-2.

The benefits of remote sampling The effect of lockdowns and isolating of the most vulnerable within our populations meant that many people were ordered to stay at home as much as possible. As a result of this, and in order to understand the serological landscape pertaining to COVID-19, institutions had to think creatively in order to run seroprevalence studies safely. This led to an urgent need for effective and precise remote blood collection devices and given the quantitative nature of many seroprevalence tests, volumetric collection became of interest as a good approach. The reason for this is that without accurate volume collection, bridging between serum and dried blood would be problematic. For example, collection using dried blood spot (DBS) often relies on sub-punching a fixed diameter disk from the sampled card. It has been shown in previous studies that biases have been seen in analytical data as a result of the viscosity of the blood which is related to hematocrit (HCT) content [2–4]. The higher the percentage HCT, the more viscous the blood is, and this results in a smaller diameter spot on the DBS card compared with a lower percentage HCT sample if the same volume is applied. This can then lead to a positive bias in the data from the fixed diameter sub punch. Interestingly, as antibodies are only found in the serum portion of the blood, the higher the HCT, the lower the amount of antibody that would be observed compared with the same volume of serum. As a result, it can be argued that both biases would act to cancel each other out, but this is not a satisfactory situation. Indeed, by collecting a fixed volume of blood, the spot area bias is eliminated, which means scientists only need to deal with one bias and not two. One such solution is to measure hemoglobin content from the extraction and use this as a predicate HCT marker. This approach has been reported on in the measurement of antibodies [5,6].

One such device that several research institutions turned to was a Mitra^®^ device based on VAMS^®^, which enables a volumetric absorptive approach to microsampling [7]. Mitra devices, (launched in 2014 at the ASMS/American Society for Mass Spectrometry conference), are designed to collect a drop of a biofluid, usually blood, onto a volume-limited polymeric tip (10, 20 or 30 μl) on the end of a device that looks like a pipette tip. When touching the polymer tip to the drop of blood, the tip rapidly fills (within approximately two seconds) using capillary forces. This straightforward method of sample collection means that practically anyone can self-collect high-quality blood microsamples anywhere.

For remote collection, the microsampling devices are contained inside secure vented cartridges, which allow for sample drying in the cartridge to reduce contamination. Once the device cartridges are placed in a foil bag with desiccant, they can dry *in situ* during transport to a lab for analysis. As with some other dried blood devices, such DBS, many analytes have been found to remain stable in dried blood, reducing the requirement for costly cold chain shipping [5].

The work described within this review summarizes how Mitra has been utilized by researchers to obtain samples, often remotely and sometimes self-collected by participants. The review is split into three main parts, the first and largest section is focused on how Mitra was used to help understand the degree of seropositivity in medium and large cohorts. A common theme seen throughout this section is that the scale of COVID-19 infection within populations had been underreported. This is because when organizations had previously relied only on PCR to measure incidences of COVID-19, not all cases were detected. This is because when individuals had recovered from COVID-19, presence of the antigen would tend to clear from the body. However, antibodies raised against the coronavirus can remain detectable many weeks, if not months, postconvalescence.

As the pandemic continued, understanding the way the immune system reacts to SARS-CoV-2 infection, was critical in terms of immunokinetics and also in terms of being able to distinguish between natural and vaccine-induced immunity. As a result, the second section of this review reports on how studies using remote blood microsamples have aided this, showing for example that there is a positive correlation between the severity of disease and longevity of antibody response.

The final section of this review looks at how Mitra devices have been used in clinical trials of COVID-19, including those that measure drug levels of possible drug candidates. Indeed, prior to the pandemic, there had been an increasing desire to decentralize clinical trials and promote trial participation from home. It was hoped that giving patients a choice about where samples were collected would help improve recruitment, compliance and retention. Indeed, for some cohorts, sampling in the comfort of the home environment would provide a number of benefits. Mitra devices have been used in a number of clinical trials in the past with success, so it seemed logical for such devices to be used in clinical trials for COVID-19. Furthermore, due to national lockdowns, with less people willing or able to attend a clinic, remote sampling was one of the more attractive and practical options to allow for the continuation of clinical trials.

Although there are a number of benefits to remote sampling, as outlined in the work reviewed, because samples from Mitra are limited in volume, care must be taken to adjust dilutions or seropositivity limits to compensate for the reduced sample input volume. Furthermore, for many analytes such as immunoglobulins, negative biases between whole blood from Mitra and venous serum samples have been observed. This is because immunoglobulins are found primarily in the serum fraction of the blood and much less so in the red blood cell fraction. As a result, researchers have used tools to compensate for these biases.

It must be noted that due to the fast-moving pace of research into COVID-19, a number of the references quoted in the manuscript are preprints and thus, upon review, data and its interpretation may change in due course. Nevertheless, it can be seen from the research outlined in this review that Mitra has been shown to be an important tool in the battle to fight COVID-19.

## Analysis of antibodies from dried mitra samples

### Prepandemic studies using VAMS technology

In 2019, 1 year prior to the coronavirus pandemic, a team led by M Zand, demonstrated that blood samples could be reliably collected using Mitra devices for a serological study they were conducting to monitor 33 strains of influenza using a multiplex immunoassay [6]. The team recruited 20 healthy volunteers from the New York area, where they collected both venous blood and capillary blood using Mitra devices in the clinic. The samples were analyzed in the laboratory to compare IgG levels in serum derived from venous blood and the dried capillary Mitra microsamples. Additionally, the volunteers were sent home with self-collection kits containing Mitra devices. They were asked to self-sample and post the samples back to the lab using either express mail (2 days) or regular mail (5 days). These self-collected samples were then compared with the staff-collected in-clinic samples. The research group showed there was a high level of concordance between serum and staff-collected Mitra samples (R^2^ = 0.9721; p < 0.0001). They reported that an equally high concordance was observed between the self-collected Mitra samples and those collected onsite by professionals (R^2^ = 0.9796; p < 0.0001). The dried blood samples were also found to be stable for up to three weeks at room temperature (22–25°C) where 94.5% of the activity was observed compared with the controls. It must be reported, however, that a significant loss of activity was observed after 28 days in storage under the same conditions. It bears mentioning that the study was conducted in the New York area in August, where temperatures can reach around 30°C and possibly even hotter in mail vans. Nonetheless, the remotely collected samples safely arrived at the lab through simple transport without influencing results. This led the group to conclude that the remote sampling approach would ‘enable subject enrollment, consent, and sample collection across large populations and disparate geographic areas.’ They proposed that the remote method could also be used to assess efficacy of the seasonal flu vaccine.

Indeed, back in 2018, a research group in the Netherlands also reported on excellent extraction efficiency and sample stability in a study of monoclonal antibody drugs [8]. The Netherlands study showed stability on Mitra microsamples comparable to the stability shown by the Rochester study. This was the second of two papers which were published that year on monoclonal antibodies from Mitra extracts. Indeed, Li *et al.*, publishing in the journal *Bioanalysis*, successfully validated a preclinical assay to measure trastuzumab and daclizumab. They concluded that this microsampling approach improved the 3Rs (reduction, refinement and replacement) of preclinical drug development [9]. These early studies provided evidence of the efficacy of using Mitra devices as vehicles for the remote collection of blood samples for measurement of intact antibodies.

### Serological targets to measure raised antibodies against coronaviruses

When a virus attacks, the immune system mounts a counterattack on the invader through interplay of the innate and adaptive immune systems. The innate immune system is inherited, nonspecific and is the first line of defence. The adaptive immune system utilizes several tools to mount a specific attack on a new invader or on a microorganism that evades the innate defences. One tool that the adaptive immune systems use is in the production of specific antibodies raised against the invader organism. These glycoproteins have several functions to help the body fight off the invader. For example, they can act as flags for other components of the immune system to destroy the attacker. They can also block the microorganism from attaching to specific receptors. In the case of the SARS-CoV-2 virus, antibodies block it from attaching to the ACE-2 receptor of the epithelial cells of the lungs, thus preventing cellular invasion [10].

There are three classes of antibody involved with viral infections and these are IgA, IgM and IgG. IgA and M are produced early on in infection, while IgG develops later on and typically has a longer half-life. All three classes of antibody can be raised against many of the structures found on viruses. In terms of SARS-CoV-2, the three target structures important for serological detection are nucleocapsid (NC), Spike protein (S) and receptor binding domain (RBD). The reason for this is that they induce high yields of antibody specific to the SARS-CoV-2 virus antigen. The NC protein is associated with the viral RNA, the S proteins are structures which protrude out from the sphere of the SARS-CoV-2 (as well as other viruses), and the RBD protein is located on the terminus of the S protein. As the name suggests, the RBD binds to the host receptor (the ACE-2 receptor, in the case of the SARS-CoV-2).

For the serological studies outlined below, one or more of the target antigens were tested against one or more antibody classes.

### COVID-19 serosurveillance using remotely collected samples

#### The National Institutes of Health conducted a nationwide serosurveillance study

In April of 2020, a research team at the National Institutes of Health (NIH) led by K Sadtler, announced a SARS-CoV-2 seroprevalence study involving around 10,000 volunteers to quantify undetected cases of COVID-19 infection [11]. The majority of the volunteers participated in remote, at-home sampling using Mitra devices. In a recent article published in Nature Communications, the team discussed their study design and proof-of-concept data [12]. The study demonstrated the development of a semi-automated, robust ELISA assay, which could be easily implemented in other labs using common equipment and reagents. It was the intention of the group that their assay design could be replicated in other labs using common equipment and reagents to run similar serosurveillance studies.

The assay was developed on both spike and RBD focusing on IgG, IgM and IgA antibodies, with an estimated specificity of >99%. The assay showed minimal cross-reactivity for spike proteins of other coronaviruses (MERS, SARS1, OC43 and HKU1) and no cross-reactivity for anti-influenza A H1N1 HA1. The research group validated their assay on both serum, derived from venous blood, and dried blood collected on Mitra devices. By developing a matrix-specific protocol, the authors obtained a strong linear correlation between serum and dried blood-derived results (R2 spike = 0.992, R2 RBD = 0.961), thereby demonstrating the suitability of the method for both matrices. Furthermore, the assay was shown to be reliable, and the team reported that ‘quantification of antibodies from mail-in sampling devices was possible.’

The preliminary results of the NIH study were published on the medRxiv website in a report entitled, “Mapping a Pandemic: SARS-CoV-2 Seropositivity in the United States.” [13]. Out of 462,949 self-selected volunteers from across the United States, a total of 11,283 completed the study enrollment, and 9089 returned both their Mitra microsamples and the survey. Only 61 of these study volunteers failed to appropriately self-collect a sample, and 971 failed to fully complete the survey. Nevertheless, the research team was able to analyze the data from 8058 volunteers. This allowed them to estimate seroprevalence across the US, while also looking at the demographic and socioeconomic differences in the prevalence of SARS-CoV-2 asymptomatic infection. The main conclusion from this data-rich dataset, was that there were approximately 4.8 undiagnosed cases (95% CI 2.8–6.8) for every reported COVID-19 diagnosis. From these data, the researchers were able to estimate that by mid-July 2020, there were 16.8 million undiagnosed COVID-19 cases in the United States.

#### Findings from a study out of Essex County, NJ, USA

In the fall of 2020, a group of researchers at Rutgers University ran a serosurveillance project similar to the one at NIH, but the Rutgers team focused on the Essex County, New Jersey (USA) area. Using a venue-based study (e.g., sample collection at shopping centers), 1349 individuals were approached for screening. Of those, 924 consented to complete a survey and have a Mitra microsample taken to be analyzed in laboratories at Rutgers University. Like the NIH, the Rutgers team developed an ELISA focusing on IgG raised antibodies against the RBD of the SARS-CoV-2 spike protein. They concluded that this would allow them to predict protective immunity, as the RBD was the main target for neutralizing antibodies.

The key findings of the study suggested that the true burden of SARS-CoV-2 of those potentially spreading the virus was reported to be more than six-times what had been previously estimated by antigen testing through PCR. These data included both symptomatic and asymptomatic subjects. Furthermore, out of the total pool measured, the researchers estimated that the number of asymptomatic persons may be close to 1.5 × greater than those who were reported to be symptomatic. Another observation the team reported was that at least 9% of the study population showed some protective immunity. One limitation raised within the study was that the demographic of Essex County is predominantly Black or Latino and so it was recognized, in the paper, that the study oversampled these groups, risking a weighting in the data [14].

#### The COVID-19 community research partnership

Mitra devices are being used to understand degrees of seropositivity by the COVID-19 Community Research Partnership coordinated by Wake Forest University School of Medicine. At the time of writing, the group has published two manuscripts exploring both degrees of seropositivity and reversion measured in the populations tested.

The first publication was focused on a longitudinal study in which more than 30,000 serological samples were taken from 11,461 adult volunteers from Wake Forest Baptist Health and the Atrium Health systems. Within this cohort, a subset of 4313 Mitra microsamples were mailed in and analyzed using a lateral flow assay (LFA) in the laboratory. The study, which ran from April 2020 to January 2021, indicated a crude seroprevalence of 10.2%. Interestingly, the estimated time for 50% seroreversion was 35.7 days. This was not associated with age, sex, race/ethnicity, site of enrollment, or whether they were a healthcare worker or not. The study found that seroreversion was significantly faster for those who were asymptomatic or had very mild symptoms. This finding led the authors to comment that cross-sectional COVID-19 serosurveillance studies have underestimated the population prevalence prior to infection. Also, the team raised the question about long-term immunity for those who had had little to no symptoms [15].

The second publication from the COVID-19 Community Research Partnership focused on analyzing 49,000 serological results from 17,000 participants in the North Carolina region [16]. The purpose of the study was to provide an estimation of infection rates or presence of antibodies to SARS-CoV-2 over the duration of the study (Apr 2020–Feb 2021). The majority of the cohort were sent specimen collection kits with lateral flow (LF) immunoassay. They were able to self-report using a mobile device application. Because this was a longitudinal study, an average of 3 (±1.9) serology tests were conducted per person over the duration. As with the previous study, a subset of specimen samples was collected on Mitra devices and mailed to the lab for analysis (part of the study control). The cohort who participated were from a mixed demographic, with 35.2% being healthcare workers and 33% being female. There were two main date ranges which the group reported on. The first was the degree of seropositivity prior to any vaccination (before 20 December 2020). The second was seropositivity at the end of the study (February 2021), after a vaccination had been rolled out for healthcare workers and those who were over 65 years old.

The findings of this second paper from the partnership showed that prior to December 20, the probability of seropositivity was 32.6%. In contrast, a March 3 report from the North Carolina Department of Health and Human Services, indicated a cumulative incidence of 8.3% total infections [17]. The reason for the disparity, according to the Research Partnership report, was that the Department of Health and Human Services data had been based on PCR and antigen tests, rather than on serology tests. Indeed, similar under-reporting levels had occurred in other studies where antigen testing had later been compared with prevalence of seropositivity [10,11]. This led to conclusions that serology gives a more accurate predictor of the rates of infection both past and present compared with direct antigen testing. The reason for this is that, if successful, the body will clear the viral antigen and RNA [18] whereas the duration of seropositivity can be observed many months after convalescence [19]. It must be noted that due to effects of seroreversion, as highlighted in the first paper from the Research Partnership [13], the total population prevalence still may be underreported.

Another key finding in the second paper from the COVID-19 Community Research Partnership was the positive effect of vaccination on the population. The researchers reported that 83% of healthcare workers and 49% of nonhealthcare workers were seropositive after vaccination, compared with 32.6% prior to vaccination. However, it was noted that this may not fully translate to immunity. Therefore, it was reported that more work is needed, such as analyzing hospitalization rates, and vaccination efficacy against variants, to fully understand the immunity landscape. However, it has been previously calculated that the herd immunity threshold to COVID-19 must be at 67% [20]. If seropositivity rates do translate to immunity, then immunization gives us hope that the >67% herd immunity threshold could be achieved. There have been some very positive signs of a reduction in COVID-19 cases, hospitalizations and deaths in the UK, as reported by the government. As of early April 2021, more than 45% of the adult population in the UK has been vaccinated, which gives a further glimmer of hope that mass vaccination is having a very positive effect [21].

One of the observations of the LF immunoassay highlighted in the study was the reported accuracy of the two LF immunoassays being sometimes lower than other immunoassay techniques. The assays used were Syntron Bioresearch Inc. LFA, which was used for Mitra extracts as well as home sampling. The second LFA from Innovita Biological Technology Co was introduced used later on in the study when the previous LFA became unavailable. Although LF can be less accurate than other immunoassays, it must be noted, however, that LF is an effective tool used in remote settings due to the immediacy of data generation upon sampling. Furthermore, the lateral devices used in the study were colorimetric point of care devices that provided a visual result. Thus, they did not rely on instruments in a lab to produce data. This was not the first time that extracts from VAMS microsampling tips had been evaluated for use with LF immunoassay combining the benefits of both techniques. Indeed, in May 2020, a research group compared dried blood samples and plasma with ELISA and LF. The group found that there was 100% agreement between both techniques for IgG. For IgM, however, there was less comparability between the two immunoassay techniques [22].

It is important to note that the cohort for this study was limited, so measuring a larger population like the one reported by the COVID-19 Community Research Partnership would provide additional statistical evidence for analysis. Another potential reason for the lower concordance in the data was that the extraction time from the dried sampling devices was much shorter (10 min) than what had been previously published. In some of the other studies outlined in this review, extraction times ranged from 2 h [20] to overnight incubation [9]. Lower extraction times could lead to incomplete extraction efficiencies and so a less sensitive detection method may not pick up all seropositive samples. Finally, as reported by the COVID-19 Community Research Partnership [13], LF immunoassay, although not perfect, was probably one of the only practical methods for measuring seropositivity in cohorts of that size. Nevertheless, combining VAMS technology with lab-controlled ELISA for a large cohort study, such as the one reported by the NIH [9,10], may also provide an alternative/additional solution for future studies.

#### Adapting Emergency Use Authorization methods on clinical chemistry analyzers

As an alternative to LF immunoassay (as used by the COVID-19 Community Research Partnership [12,13]) and the development of ELISAs (as reported by the NIH [9] and Rutgers University [11]), an approach that has shown success has been to adapt assays run on large analyzers for extracts from the VAMS sampling tips. This has the advantage of utilizing an assay that has already been validated for COVID-19 in serum. Therefore, adaption to extracts from dried blood may be easier than developing a fresh method from scratch or using LF (which can have accuracy limitations), as highlighted by the COVID-19 Community Research Partnership. Furthermore, large analyzers are designed to work accurately and quantitatively under high-throughput conditions, making them an attractive option for large population studies, especially where increased quantitation is required.

A research group at Massachusetts General Hospital adapted an Emergency Use Authorization (EUA) SARS-CoV-2 total antibody assay (Roche Elecsys^®^) to work with extracts from the VAMS sampling tips using a routine analyzer (Roche Cobas^®^ 8000 e801 Immunoassay Analyzer). By modifying the method to account for the differences in sample concentration, the group was able to show 99–100% concordance between the two matrices. The group then ran a serosurveillance study in the Chelsea, MA area, where they collected both serum and Mitra samples. Both sample matrices resulted in an identical seroprevalence of 24%. This was the first time such an assay had been developed on a large commercial analyzer for SARS-CoV-2 serology [23]. A second group led by A Marchand in France, also developed a similar assay on the same platform [24]. One observation made by the group at Massachusetts was that the Roche assay had been developed to detect total antibody concentration for IgA, IgM and IgG raised against the NC protein. It was noted that this would work well for measuring naturally raised antibodies against SARS-CoV-2 but would not be able to track vaccine-induced immunity. The reason was that vaccine developers had targeted the spike protein for their vaccine development, rather than the NC protein [25]. The authors commented that running two separate assays, one targeting spike and the other targeting NC protein, would allow groups to differentiate between vaccine-induced and natural immunity.

### Understanding immunity to SARS-CoV-2

While many groups, such as the NIH, were running studies using VAMS technology to remotely collect samples in an effort to understand the scope of seropositivity across geographic regions, other groups were running studies to understand the role of antibodies in COVID-19 infection [12,18,19,26]. Key questions have been raised around the effect the virus has on the body’s antibody response. For example, does prior exposure to other coronaviruses give any protection? Or worse, does prior exposure bring about a deleterious response to SARS-CoV-2, such as epitope masking and/or original antigenic sin [19,27]? Original antigenic sin, also known as the Hoskins effect, is the situation where the body has B-memory cells raised against a related virus with a high degree of homology to the new virus and, when infected by this new virus, the body does not react as strongly as predicted [28]. Epitope masking is where an IgG hides the antigen recognition by specific B cells, which leads to a reduced raised antibody response to a new related invader [26].

#### Findings from an immunokinetic study of SARS-CoV-2 in New Zealand

Some of the questions posed by researchers investigating the immunology of COVID-19 has been around the degree to which raised antibodies are formed: what classes are they, what is their longevity, and which are neutralizing?

A group of researchers led by Associate Professor N Moreland in New Zealand ran a longitudinal study where they used a multiplex immunoassay to measure antibodies (IgA, IgM and IgG subtypes 1, 2, 3 and 4) raised against spike, RBD and NC proteins. They also investigated which of these antibodies might neutralize SARS-CoV-2 [29]. At the time that the study was carried out, New Zealand had practically eliminated SARS-CoV-2 from its island nation. Their unique geography gave the group an opportunity to study longevity of immunity, where repeat infection rates were extremely rare, so there was almost no risk of immune boosting in their environment. This allowed the group to predict the duration of raised antibodies, to determine which antibodies were neutralizing, and which protein stimulated the immune system to provide the longest protection. Using a cohort of 113 prepandemic negatives and 189 COVID-19 positives, the group showed that 99% of sera had anti-RBD IgG, and 96% had anti-S protein IgG levels that were above baseline levels between 4 and 8 months post infection. Interestingly, the majority of the volunteers had experienced nonsevere infections.

As expected, levels of IgA and IgM returned to baseline a lot quicker than IgG antibodies. Indeed, for IgM, over three quarters of the samples showed a return to baseline raised for all three antigens during late convalescence. In terms of neutralizing antibodies, anti-RBD IgG were still showing around 90% inhibition more than 124 days after symptom onset. Using three statistical models, the researchers were able to estimate a half-life of neutralizing IgG of 5–20 months. Finally, the group demonstrated excellent correlative data when comparing sera to dried extracts from the Mitra sampling tips (n = 19) on a subset of samples measuring IgG antibodies raised against Spike (R^2^ = 0.9918; p < 0.0001), RBD (R^2^ = 0.9929; p < 0.0001) and NC (R^2^ = 0.9957; p < 0.0001). The group concluded that using dried fingerprick blood samples would provide feasibility in future immunokinetic studies of SARS-CoV-2 highlighting the importance of tracking vaccine responses in a population with low or no SARS-CoV-2 in circulation.

#### Discrimination of SARS-CoV-2 natural & vaccine-induced immunity & the role of pre-exposure to other coronavirus infections

Multiplex immunoassay to examine antibody reaction to SARS-CoV-2 was also employed by a group headed up by ED Lang and CC Broader as part of the Infectious Disease Clinical Research Program (IDCRP) in the United States. The IDCRP has a number of ongoing projects investigating ways to monitor and fight COVID-19. One such study is the EPICC (Epidemiology, Immunology and Clinical Characteristics of Emerging Infectious Diseases with Pandemic Potential). For this longitudinal project, military personnel in the US is being tested using Mitra devices to measure the serological responses of COVID-19. The aim is to help improve clinical care and management of those with the disease and inform future prevention of such diseases [30].

Recently, the IDCRP group has published their first study examining the serological impact of previous exposure to other coronaviruses, such as SARS, MERS and both α and β-coronaviruses (α-Cov and β-Cov), to SARS-CoV-2 infection [31]. Using a multiplex immunoassay approach and through creating three bead mixes, the group was able to record (using the first bead-mix) the effect of pre-exposure to β-Cov (including SARS, MERS and two human coronaviruses [HCoV]) on the serological response of SARS-CoV-2. The second bead mix was used to examine the effect that both α and β-HCoV (four strains in total) had on SARS-CoV-2 infection. The third bead mix was developed to examine natural versus vaccine-induced immune response to SARS-CoV-2 antibodies raised against NC and spike protein, respectively.

Using three cohorts, the first (Acute Respiratory Infection Consortium Natural History Study [ARIC]) was taken from prepandemic samples, and the second from the above-mentioned EPICC, involving a sample group who were all COVID-19 positive with a range of disease severity and not hospitalized (where Mitra was used to collect remote blood samples). The final cohort (hospitalized patients at the Javits Medical Station [JMS]) involved a COVID-19 positive group of people who were hospitalized with more serious symptoms (where serum samples were used). The findings showed that in people with more serious COVID-19 (JMS), IgG levels for SARS-CoV-2 (Spike and NC) were higher than in those with low to moderate category illness (EPICC). A similar trend in the cross-reactivity to β-Cov was also observed. The researchers observed a similar trend with the HCoV bead mix, too.

Interestingly, using nonhuman primates (no prior exposure to HCoV), no cross-reactivity was observed when these subjects were infected with SARS-CoV-2. Another observation showed that with enough heightened inflammation, cross-reactivity can even be seen with human α coronaviruses, which are distantly related to SARS-CoV-2. De-novo cross-reactivity with SARS and MERS-CoV from SARS-CoV-2 positive cohorts demonstrated that there were some shared epitopes, which led the group to suggest evidence of a possible target for pan-zoonotic vaccine development. However, they reported that they did not find that evidence of pre-exposure to HCoV OC43 provided any back-boosted responses. Moreover, pre-exposure seemed to have a negative production effect on neutralizing antibodies.

Combining serology and direct antigen measurement to understand both the kinetics of SARS-CoV-2 infection and raised antibodies from dried blood, In March 2021, a team led by D Shan, reported that they employed a high-sensitivity single-molecule array immunoassay (Simoa^®^) platform to directly measure the NC protein in both saliva and on Mitra microsamples [18]. One of their goals was to improve upon the existing methods available for diagnosing COVID-19 illness. An existing method for diagnosing COVID-19 is PCR measurements from nasopharyngeal swabs. However, this is an uncomfortable sampling procedure, and a number of reports also have highlighted the risks of false negatives, especially within the first 6 days of symptom onset [32,33].

An alternative method for COVID-19 diagnosis is to conduct direct antigen testing using immunoassay. One such assay, using the Simoa platform, has recently received EUA from the US FDA. This EUA is for nasopharyngeal swabs demonstrating a 97.7% positive percentage agreement (PPA) compared with PCR [34].

With several studies demonstrating the role that plasma viremia has on COVID-19, and its possible predictor of mortality [18,35,36], D Shan and team embarked on developing a sensitive direct antigen test to measure the viral NC protein for both dried blood Mitra microsamples and also saliva [37]. Comparing to nasopharyngeal PCR, their assays showed >90% PPA for SARS-CoV-2 positive patients, and >98% negative percent agreement, for all matrices within a week of a PCR+ test. The team also developed an immunoassay on the Simoa platform to measure IgG response to the spike protein. By using both antigen and antibody assays from the same sample, they were able to investigate the kinetics of SARS-CoV-2 infection. In short, the team was able to demonstrate an inverse relationship between the antigen and IgG raised from the spike protein.

Using the Mitra devices, the team collected 62 samples from staff and residents of a long-term care facility and compared these to PCR samples from an FDA-authorized assay. They demonstrated 100% negative percent agreement and PPA for days 1–7. Furthermore, when ranking the PCR+ donors, any increasing N-protein level was compared with disease severity, which demonstrated that worse disease severity was associated with higher N-protein level. The team acknowledged, however, that there were limitations to their assay, such as the size of cohort. While further work is needed, Shan *et al.* showed the utility of combining both antigen and antibody tests on dried capillary blood samples collected from the same cohort. Their work further illustrated that this would be a useful method, not only for tracking the kinetics of SARS-CoV-2 infection, but also for expanding tools to detect active SARS-CoV-2 infections without resorting to uncomfortable nasopharyngeal swabs. Furthermore, as viral RNA can remain detectable post infection, measuring kinetics of antigen levels with increasing antibody titres would be more of a challenge if PCR were employed rather than antigen detection in blood.

#### Using microfluidic nano-immunoassay as a future tool for SARS-CoV-2

As the coronavirus pandemic enters its second year, much has been learned about the immunopathology of COVID-19 disease. We have also learned that tools like portable Mitra devices have enabled research groups to conduct large, decentralized population studies to understand seropositivity, immunokinetics and more. One bright light in the battle to fight COVID-19 has been the remarkable development of fast-track vaccines, which are helping societies gain wider immunity and slowly ease up on their social distancing restrictions. There has also been more discussion of immunity and vaccination passports, which might allow people to safely resume travel.

As a result of these developments, a partial switch from antigen and PCR testing to immunity testing may be on the horizon. Indeed, as far back as April 2020, the WHO published a scientific brief on the subject [38]. If immunity testing becomes commonplace, then there could be a significant upswing in serology testing. This would make very high throughput assays an attractive tool for coping with a huge influx of blood tests. As the newer threat of SARS-CoV-2 variants spread through populations and cause new infections that cannot be prevented by current vaccines, continued serosurveillance may be required.

One solution to increase assay throughput would be to miniaturize the immunoassay using microfluid chips. With this approach, hundreds of samples (with optimization, conceivably thousands of samples) could be analyzed from one device. In fact, a team from Switzerland has demonstrated the utility of combining microsamples from devices such as Mitra and interfacing the extracts on a microfluid chip the size of a USB stick [39]. Due to the miniaturization of the assay, the group commented in their paper that this would be a far less expensive method compared with, for example, LF assays that typically cost about $22 per test. Using 134 negative and 155 positive control sera, the group reported a specificity of 100 and 98% sensitivity when PCR was used as a reference. The team demonstrated that the assay outperformed a reference ELISA, with the chip showing upwards of 33% more positives than the traditional technique. The team hypothesized that a small team of three technicians could conceivably process 3072 samples per day from just six devices.

## Using VAMS in COVID-19 clinical trials

There are a number of approaches researchers are exploring internationally to find effective COVID-19 treatments. One approach is to use antiviral therapies, such as the drug remdesivir, which has been shown to provide benefits in treating COVID-19 disease [40]. Other therapies being investigated are those that target the inflammatory pathway, including steroids such as dexamethasone propionate, which have proved to be effective [41]. Monoclonal antibody drugs such as the IL-6 inhibitor tocilizumab, have shown promise. Recently, however, there was a report that tocilizumab does not, unfortunately, significantly improve outcomes for patients with severe COVID-19 pneumonia [42]. Another therapy which has given unclear results is convalescence plasma therapy [43], where immunoglobulin rich plasma from a newly recovered patient is donated to a recipient in the hope that the antibodies in the plasma will help to fight the virus.

At the time of writing, clinicaltrials.gov lists more than 3400 global clinical trials focused on COVID-19, which are just launching or are already recruiting study volunteers [44]. One drug, which has gained notoriety in the popular press, is the antimalarial drug hydroxychloroquine (HCQ). However, it has been largely concluded that HCQ is ineffective and this drug was reported to show (at best) mixed results, according to a recent review by J Majumder and T Minko [45]. Nonetheless, HCQ may still have potential as a COVID-19 treatment, and there are currently 152 active or recruiting trials further evaluating the drug, either in isolation or in combination with other treatments [46].

Prior to the COVID-19 pandemic, remote sampling using Mitra devices was beginning to gain good traction, with a number of clinical trials reported in the literature. Sixteen of these trials were listed in a recent review by Y Harahap and colleagues [47]. Additionally, a paper has recently been published by AstraZeneca, discussing the benefits of patient-centric sampling using Mitra devices (and other remote solutions) to allow for a mixed approach to sample collection for clinical trials. It was proposed in the publication that by giving study volunteers a choice of sample collection, either in-clinic collection by a trained professional or self-collection at-home (or both), researchers can design a hybrid approach to trials that will improve recruitment and compliance [48].

Since the pandemic, the interest in remote specimen collection has increased significantly. This is because, unsurprisingly, most people are no longer willing to travel to clinical trial facilities for routine visits. Trial-related travel and in-clinic processes were always viewed by volunteers as barriers to participation, but these inconveniences are now considered risky, as well.

During the pandemic, a clinical trial was reported where Mitra devices were used for remote blood collection to test the efficacy of the above mentioned antimalarial drug HCQ as a prophylactic against COVID-19. VAMS technology had previously been used in a published HCQ study where remote sampling with Mitra devices was offered as an option to rheumatoid arthritis patients. The patients in that study reported a more successful collection experience compared with DBS cards, where 20% of the cards were unable to be completely filled [49]. The same research team also has recently reported that the drug HCQ remains stable on Mitra devices for up to 5 years [50]. Due to the prior success in measuring samples from Mitra, as part of the COVID-19 HCQ prophylactic trial, blood measurements were taken from healthcare workers using Mitra devices [51]. The trial ultimately showed that HCQ was ineffective as a prophylaxis, but the trial demonstrated how effective remote sampling was as a tool for enabling clinical trials to proceed during the additional barriers and difficulties imposed by a pandemic.

## Conclusion

The COVID-19 pandemic took the world by storm, but a remarkable effort by the global medical and scientific communities has fast-tracked our understanding of the pathology of SARS-CoV-2 and COVID-19. New discoveries include effective treatments, such as dexamethasone propionate, which have reduced death rates of COVID-19 patients by a third as compared with standard supportive care [39]. The remarkable speed with which effective vaccines for COVID-19 were developed and deployed is illustrative of how modern medicine and science can deliver in difficult and unprecedented circumstances. Although the world is not out of the woods, our understanding of how to treat and vaccinate against COVID-19 is increasing. We have also learned that tools for remote sampling of blood using Mitra devices with VAMS technology is helpful for decentralized or remote immunological studies and for treatment efficacy studies. Indeed, there are many remote microsampling studies currently underway or being proposed that aim to further improve our understanding of the immunology of COVID-19. Some remote studies are measuring inflammatory responses to the virus, while others are tracking vaccine efficacy. A growing body of studies indicate that remote sample collection and dried matrix analysis have become ‘go-to’ techniques in the battle against COVID-19.

## Future perspective

The COVID-19 pandemic brought into focus how a globalized society reacts to such a world-changing situation. There have been many bumps in the road, with limited supplies of Personal Protection Equipment and some concerns about access to tests for some countries. Additionally, treating hospitalized patients with severe COVID-19 has been a long learning curve for healthcare professionals. However, as the healthcare and science communities have gained a deeper understanding of SARS-CoV-2 infection and how to treat it, outcomes are much better now than they were at the beginning of the pandemic. A huge and unexpected positive that has resulted from the COVID-19 pandemic is the remarkable speed with which we have increased our understanding of the serological landscape. This has led to rapid vaccine production and, critically, deployment. At the time of writing, the United Kingdom is predicted to soon gain herd immunity, roughly 12 months after the first national lockdown. If the UK meets its herd immunity goal, this will be an unprecedented achievement in the nation's history. It is encouraging that this is being replicated in many countries globally, such as Israel and the USA.

The pandemic has demonstrated that the near impossible is achievable. For example, remote blood collection using volumetric microsampling techniques such as VAMS has proven to be an effective weapon in the pandemic response. The remote microsampling approach has allowed researchers to gain a clear understanding as to the impact of the virus on societies, as well as gain insight into the immunogenetics of the response to infection. Furthermore, it has allowed data to be gathered from rapidly deployed clinical trials in difficult to reach cohorts.

Remote microsampling has delivered samples which have been demonstrated to be as accurate as traditionally collected in-clinic samples. This alternative approach to blood collection proved to be critical where traveling to clinics became risky, and in some cases, not possible. The pandemic has shown that remote microsampling can be an effective equivalent to traditional venous blood draws. Furthermore, it has shown the potential for widespread adoption of new options for patient diagnosis and monitoring. It also has shown that clinical research can be effectively achieved remotely, using telemedicine coupled with remotely collected samples. This approach removes geography from the equation. It is now conceivable that home-collected samples can be sent across the world without the need for costly cold-chain shipping. This gives more people access to the best laboratories in the world, independent of location.

It must be noted however that microsampling is not without its drawbacks. The first is that the small samples collected are not always compatible with certain lab instruments. For example, clinical chemistry analyzers often require a minimum sample input volume, typically around 100–300 μl (dependent on the instrument). Furthermore, special adapters are often required to work with such low volumes and so can impede throughput. Moreover, the analyzers are often ‘closed systems’, designed for analyzing a specific undiluted biofluid, where no modification to the method is possible. Extracts from dried microsamples are diluted, often around 10–20-times, which may be too dilute a matrix for the analyzers to measure. This can be addressed, however, if the analyzer has the option to run in an ‘open’ mode where any ‘in-instrument’ dilutions can be avoided. These ‘open’ options on such instruments mean that the methods developed/modified would be research-use-only, so a laboratory development test would need to be validated to governmental guidelines.

Another drawback to using dried blood microsamples is that drying the blood and subsequent rehydration causes total hemolysis of the sample. This prevents certain assays from being analyzed from such solutions. This is the case if the analyte of interest is found in abundance in the HCT, but not in the plasma/serum fraction. An example of this is potassium, where the test measures trace amounts of the element in serum. Other tests that are less compatible with dried blood extracts are colorimetric and homogeneous in nature and require clear biofluids, such as serum, to allow for sensitive spectrophotometry. A fully hemolyzed sample often risks too much interference in the measurement. An example of such a test is the measurement of liver enzymes, such as alkaline phosphatase, in serum. Finally, blood is composed of typically around 40–45% HCT. This means that if analytes are found only in the serum fraction of the blood, as is seen with antibodies, negative biases are often seen when comparing extracts from dried blood. Indeed, as discussed in this review, scientists have used a variety of techniques to compensate for the presence of HCT. The NIH assumed 50% reduction in signal for all samples and adjusted accordingly [14]. M Zand’s group measured hemoglobin in the dried microsamples to arrive at a predicted HCT level and corrected each datapoint to arrive at an estimated serum level [9]. Finally, Massachusetts General Hospital, using a clinical chemistry analyzer, re-established seropositivity limits to compensate for the presence of HCT and the prediluted sample [25].

Thankfully, analytical technologies such as multiplex immunoassay and LC–MS/MS continue to improve, so an increasing variety of analytes can be reliably measured from a single drop of blood. And as targeted longitudinal clinical tests using omics platforms, for example, begin to gain traction, they may in the future, supplant traditional standard clinical assays based upon population reference ranges. For future science and medicine, the move to remote sampling could be as commonplace as the adoption of the mobile phone was to personal communications.

**Executive summary**

- The SARS-CoV-2 pandemic and widespread COVID-19 infections required the need for remotely collected blood samples as a result of travel restrictions and social distancing for safety.
- VAMS^®^ technology enables a volumetric absorptive approach to microsampling, and is a proven technique to reliably and remotely collect high-quality samples that can be posted to laboratories for analysis. Prior to COVID-19, Mitra^®^ devices based on VAMS technology had been successfully used to measure flu antibodies from home-collected samples. This early success made VAMS technology an ideal choice for remote blood collection to help understand SARS-CoV-2 infection and to treat COVID-19.
- Groups such as the National Institutes of Health, Rutgers University, the COVID-19 Community Research Partnership and Massachusetts General Hospital have successfully run serosurveillance studies using remotely collected Mitra microsamples. All groups report overwhelming evidence that prevalence of SARS-CoV-2 infection (COVID-19) was much higher compared with what had been previously estimated for the populations tested using antigen-based testing.
- Groups such as the University of Auckland and Infectious Disease Clinical Research Program at the Uniformed Services University have used samples, including those collected with Mitra devices, to profile immunoresponse in SARS-CoV-2 infection. This led to reports of protective immunity ranging between 4–8 months post convalescence. The impact that a prior exposure to pre-SARS-CoV-2 coronavirus infection has on antibody response has also been investigated.
- Use of volumetric absorptive microsampling in measurement of antigen levels in viraemic samples led to an observed inverse relationship between blood antigen levels and antibody response in a care home setting.
- Reports on combining remote sampling with miniaturized chip-based immunoassay to improve throughput and reduce costs compared with traditional immunoassay show highly promising results.
- Use of Mitra devices with VAMS technology in COVID-19 clinical trials demonstrated that remote sample collection in populations with limited access to venepuncture was particularly helpful. Remote microsampling clinical trials were reported, and in one case, this trial approach helped to rule out the drug Hydroxychloroquine as a prophylaxis for COVID-19.
